# Reactivity to smartphone-based ecological momentary assessment of depressive symptoms (MoodMonitor): protocol of a randomised controlled trial

**DOI:** 10.1186/s12888-016-1065-5

**Published:** 2016-10-21

**Authors:** Wouter van Ballegooijen, Jeroen Ruwaard, Eirini Karyotaki, David D. Ebert, Johannes H. Smit, Heleen Riper

**Affiliations:** 1 Section Clinical Psychology, Vrije Universiteit Amsterdam, Van der Boechorststraat 1, 1081 BT Amsterdam, Netherlands; 2 Department of Psychiatry, VU Medical Centre/GGZ inGeest, Amsterdam, Netherlands; 3 Friedrich-Alexander-Universität Erlangen-Nürnberg, Erlangen, Germany

**Keywords:** Ecological momentary assessment, Experience sampling, Assessment reactivity, Smartphones, Mobile health, Depression, Mood

## Abstract

**Background:**

Ecological momentary assessment (EMA) of mental health symptoms may influence the symptoms that it measures, i.e. assessment reactivity. In the field of depression, EMA reactivity has received little attention. We aim to investigate whether EMA of depressive symptoms induces assessment reactivity. Reactivity will be operationalised as an effect of EMA on depressive symptoms measured by a retrospective questionnaire, and, secondly, as a change in response rate and variance of the EMA ratings.

**Methods:**

This study is a 12-week randomised controlled trial comprising three groups: group 1 carries out EMA of mood and completes a retrospective questionnaire, group 2 carries out EMA of how energetic they feel and completes a retrospective questionnaire, group 3 is the control group, which completes only the retrospective questionnaire. The retrospective questionnaire (Centre for Epidemiologic Studies Depression scale; CES-D) assesses depressive symptoms and is administered at baseline, 6 weeks after baseline and 12 weeks after baseline. We aim to recruit 160 participants who experience mild to moderate depressive symptoms, defined as a Patient Health Questionnaire (PHQ-9) score of 5 to 15. This study is powered to detect a small between-groups effect, where no clinically relevant effect is defined as the effect size margin −0.25< d <0.25.

**Discussion:**

To our knowledge, this is the first study to investigate whether self-rated EMA of depressive symptoms could induce assessment reactivity among mildly depressed individuals.

**Trial registration:**

Netherlands Trial Register NTR5803. Registered 12 April 2016. http://www.trialregister.nl/trialreg/admin/rctview.asp?TC=5803.

## Background

Daily, repeated measurements of mental health symptoms, also known as experience sampling or ecological momentary assessment (EMA), enables clinicians, researchers and patients to monitor psychological processes in real time. EMA is usually operationalised as active monitoring, in which patients respond to prompted or self-initiated items or questions [1]. EMA involves repeated self-report assessments of a current state, such as daily assessments of mood. Several advantages of active EMA for assessing mental health problems, such as depression, have been noted [2]: absence of recall bias; sensitivity to mood fluctuations [3]; and real-time insight into treatment response, which can also be used as feedback to individual patients (e.g. [4]). Several studies have employed smartphones for EMA of depressive symptoms (e.g. [5–9]).

Active EMA may not only measure, but also influence mental health symptoms, which is known as assessment reactivity. Studies on alcohol abuse interventions show that repeated assessments draw attention to the monitored behaviour, which can identify problematic behaviour and highlight personal responsibility [10]. In that sense, EMA is similar to a brief intervention and can have a positive influence on treatment outcome [11]. Another type of reactivity is response fatigue, which is a declining response rate or declining response accuracy over time (e.g. [12]). A declining response accuracy could be observed by a declining correlation between a repeated measure and another measure that assesses a theoretically associated construct [12].

In the field of depression, EMA reactivity has received little attention. Kramer et al. [13] found that EMA of positive and negative affect may have a beneficial effect on depressive symptoms after 7 weeks, although the effect had disappeared at the 6-month follow-up [13]. EMA was conducted alongside pharmacotherapy in that study and the EMA was short and intensive, i.e. over 10 self-rated items, each of which had to be rated 10 times a day for 5 consecutive days and for 5 more consecutive days a few weeks later [13]. Positive and negative affect are related to depression, so reactivity to EMA of depressive symptoms may follow a similar pattern as was found by Kramer et al.. Little is known about reactivity to EMA of depressive symptoms when conducted for a longer consecutive period, such as 12 weeks.

This study aims to investigate whether EMA of depressive symptoms induces assessment reactivity. First, we will investigate whether EMA of depressive symptoms during a 12-week period has an effect on depressive symptoms measured by a retrospective questionnaire. Secondly, we will investigate whether response fatigue affects the EMA ratings. Response fatigue will be operationalised as response rate over time and correlations with measures of associated constructs. To minimise response burden, participants will monitor only one symptom and 1 to 3 times a day. Because depression is a multidimensional construct [14] we will conduct EMA of two core depression symptoms in two groups, where group 1 monitors mood and group 2 monitors how energetic they feel. Based on the literature we expect a small positive short-term effect of EMA on depressive symptoms.

## Methods

### Study design

This study is a 12-week randomised controlled trial among participants who experience mild to moderate depressive symptoms. The trial consists of three groups: group 1 carries out EMA of mood and completes a retrospective questionnaire; group 2 carries out EMA of how energetic they feel and completes a retrospective questionnaire; group 3 is the control group, which completes only the retrospective questionnaire. The retrospective questionnaire is the Centre for Epidemiologic Studies Depression scale (CES-D) [15], which assesses depressive symptoms. The CES-D is administered at baseline (T0), 6 weeks after baseline (T6) and 12 weeks after baseline (T12).

### Study population and inclusion criteria

We aim to recruit 160 adult (18+) participants who experience mild to moderate depressive symptoms among college students and users of mental health websites. We define mild to moderate depressive symptoms as a PHQ-9 score of 5 to 15 [16]. All participants are required to own a smartphone that runs the Android operating system, version 4.0 or later, because the EMA application runs only on that platform. Recruitment within a student population and among website users will ensure that many of the interested individuals own a smartphone and are used to installing and using apps.

### MoodMonitor, a self-monitoring application

Participants in group 1 and 2 install the MoodMonitor application on their smartphones, which conducts EMA of mood and energy level. This app has been developed by the E-Compared consortium [8]. Every day, the participant will receive one notification on his/her smartphone at a random time point between ten o’clock in the morning and ten o’clock in the evening. This notification directs the participant to the question ‘How is your mood right now?’ (group 1) or ‘How energetic do you feel right now?’ (group 2), which the participant can answer on a visual analogue scale from 1 (worst) to 10 (best) with a precision of 1 digit after the decimal point, e.g. 8.1. The notification remains accessible until the question is answered. If a notification remains unanswered, it is replaced when the next notification is sent. The measurement will be time stamped, i.e. the system records the exact time when the participant enters a rating, in addition to the time when the notification was sent. Furthermore, participants are free to provide a rating at any time they want by opening the app. During week 1 and week 12, the participant rates his/her mood three times a day instead of just once, in order to measure mood fluctuations during the day. The entered ratings are instantly visible to the participants on a graph which they can access through the MoodMonitor application.

### Procedure

We employ three recruitment strategies. First, we distribute flyers on the university campus. Second, we post advertisements on Dutch websites for mental health issues. Third, advertisements are posted on Facebook and Twitter. The flyers and advertisements specifically target people who experience low mood or mood fluctuations and direct them to a website (www.moodmonitor.nu) that contains more detailed information. Interested individuals can apply to participate in the study via this website by completing the screening questionnaire (PHQ-9, age, possession of an Android-compatible smartphone), after which they can read the study information again and can agree to participate by entering a valid email address (electronic informed consent). Applicants who do not meet the inclusion criteria will be notified instantly. If they are excluded because they score above 15 on the PHQ-9, they are advised to contact their general practitioner. Participants who meet the inclusion criteria will be randomised equally to the three groups, i.e. 1:1:1. An independent researcher performs the allocation using a computerised random number generator. Next, participants are sent a link to the baseline questionnaire (CES-D, demographics), and participants in groups 1 and 2 receive an email with instructions to download and install the MoodMonitor app on their smartphones. Even though we do not notify participants directly as to which group they are randomised, they can find out easily by reading through the study information on the website, and therefore participants cannot be considered to be blinded. After week 6 and after week 12 participants in all three groups are sent an email containing a link to the questionnaires (CES-D at week 6, CES-D and SUS at week 12). Participants who complete a measurement are offered 7.50 Euro, i.e. 22.50 for all three measurements, and an additional 10 Euro if they respond to 80 % or more of the EMA prompts. Participants will receive this incentive in the form of an electronic gift voucher sent to their email address. We will continue recruitment until 160 applicants have been randomised. Data collection runs from April 2016 to November 2016. This trial’s results will be published on the study homepage when the scientific papers concerning the main objectives have been published.

### Instruments

Inclusion criteria will be determined using the Dutch version of the Patient Health Questionnaire (PHQ-9) [16], which will be administered online. The PHQ-9 contains nine items and covers nine criteria listed in the Diagnostic and Statistical Manual of Mental Disorders, 5th Edition (DSM-5), requiring respondents to rate the frequency of present difficulties during the past 2 weeks. Scores indicate the presence and severity of depression symptoms, with a maximum score of 27 and a minimum score of 0. Scores of 5, 10, 15, and 20 indicate mild, moderate, moderately severe, and severe depression, respectively. The internal consistency (Cronbach’s alpha) of the PHQ-9 with a clinical population was in the range of .86–.89 [17]. The instrument has shown good inter-format reliability between the original pen-and-paper and the online version [18]. We aim to include mild to moderately depressed participants, so we will include participants who score within the range of 5 to 15.

The primary outcome measure of this study is retrospectively measured depressive symptoms. We will use the Centre for Epidemiologic Studies Depression scale (CES-D) [15, 19] which will be administered online at baseline, after week 6 and after week 12. This scale consists of 20 self-rated items, each scored from 0 to 3. The total score ranges from 0 to 60, where higher scores represent more severe depressive symptoms. When used online, the CES-D has a good internal consistency (Cronbach’s alpha = .89–.93) and consists of 2 to 4 factors [20].

Several demographic variables (e.g. age, gender, educational level, marital status) will be gathered at baseline to determine the characteristics of the sample. We will also ask the participants how often and for which purposes they use their smartphones. At all measurement points participants are also asked whether they receive any professional help for mental health related problems, because that could influence their depressive symptoms.

The final questionnaire at week 12 contains the *System Usability Scale* (SUS) [21, 22] to evaluate the usability of the MoodMonitor app. Therefore the SUS will be administered to groups 1 and 2 only. The SUS comprises ten questions (e.g. ‘I would imagine that most people would learn to use this application very quickly.’) with five response options (ranging from ‘strongly disagree’ to ‘strongly agree’). Total scores range from 0 to 100, with higher scores representing higher usability. A SUS score above 70 could be considered adequate [22].

At the end of the study, we will randomly select ten participants in the EMA groups for a semi-structured interview by telephone to obtain qualitative information about: 1) their experience with tracking their mood and energy level; 2) their experience with the app and how it could be improved; 3) participation in this study in general (Table 1).

**Table 1:** Schedule of enrolment, interventions and assessments

	Study period					
	Enrolment	Allocation	Post-allocation			
Time point	*-t*_*1*_	t0	*t1-5*	*t6*	*t7-11*	*t12*
Enrolment:						
Eligibility screen	X					
Online informed consent	X					
Allocation		X				
Interventions:						
EMA of mood			X	X	X	X
EMA of energy level			X	X	X	X
Control						
Assessments:						
PHQ-9, age, Android	X					
Demographics		X				
Use of smartphones, social media		X				
In mental health care?		X		X		X
CES-D		X		X		X
SUS						X
Interviews						X

### Data management

Data obtained by the online questionnaires are stored in a password-protected database maintained by an independent data manager. The email addresses of participants, which is the only personal information we obtain, will be stored in a separate database, which is also maintained by an independent data manager and will be destroyed after data collection. Both databases are not accessible by the researchers and the data of the online questionnaires will become available for research purposes when data collection has been completed. The data obtained by the MoodMonitor app are not stored on the participants’ smartphones, but on a remote server, to which the anonymised data are sent through a secured connection. The app data (i.e. mood and energy ratings) are also not accessible by the researchers until data gathering has ended. The independent data manager monitors the data flow. There is no (other) data monitoring committee, due to the minimal burden and risk associated with participating in this study. After the publication of this study’s main results, the data obtained by this study will become available on request. Requests should be sent to research@ggzingeest.nl with the topic name MoodMonitor.

### Analyses

The primary outcome is the effect of EMA on depressive symptoms as measured by the CES-D at T6 and T12, comparing both EMA groups with the control group. To test this on all available data, we will conduct a mixed models repeated measures regression analysis, with T6 and T12 CES-D data as the dependent variable, and baseline CES-D scores, group (1, 2, 3), time (T6, T12) and the time*group interaction as independent variables. Effects will be expressed in terms of percentage of variance explained and Cohen’s d (by dividing raw regression parameter point estimates and confidence intervals by the pooled CES-D standard deviation).

For our secondary analysis, we will examine the EMA response rate over time, which gives an indication of response fatigue. Response fatigue will also be analysed by assessing response accuracy, here defined as a declining correlation over time between theoretically associated measures. Literature has shown that mood swings are associated with higher depressive symptoms [23]. Therefore, we will analyse the variance of the EMA and its correlation with the CES-D scores, taking into account the EMA ratings of the two weeks prior to the CES-D (i.e. weeks 5 and 6 for T6 and weeks 11 and 12 for T12). A decreasing variance of EMA ratings (i.e. increasingly stable responses) will result in a decreasing correlation coefficient (*r*) with the CES-D scores if the CES-D scores do not decrease. This might indicate a decline in validity.

We will maintain a family-wise two-tailed *p*-value of .05 using the Holm-Bonferroni method [24]. IBM SPSS Statistics 23 [25] and R [26] will be used for all analyses.

### Sample size

For the sample size calculation, we assume that a difference between either EMA group and the control group within the margin −0.25< Cohen’s d <0.25 is clinically negligible. Therefore, a difference between groups of d = 0.25 should reach statistical significance in our analysis. We ran a power calculation in G*Power [27] for a repeated-measures analysis of variance (ANOVA), which is essentially the same analysis as linear mixed modelling when there are no missing values. A total number of 120 participants (40 per group) is required in order to obtain d = 0.25 with three assessment waves, a targeted power of .85, a significance level alpha of .05, and conservative estimations of correlations between measurements (*r* = .65) and variances of the differences between groups (non-sphericity correction epsilon = .8). Expecting 25 % drop-out at T12, we will recruit 160 participants. All participants will be included in the linear mixed model, which is robust for missing data.

## Discussion

This study aims to investigate whether self-rated EMA of depressive symptoms could induce assessment reactivity among mildly depressed individuals. The linear mixed model analyses can show an effect of Cohen’s d >0.25 (and < −0.25) on retrospectively measured depressive symptoms between groups. The 12-week study period and repeated measures design enable us to detect both short-term (6 weeks) and longer-term (12 weeks) effects on retrospectively measured depressive symptoms, as well as response fatigue.

### Implications

This study will give a first indication of EMA reactivity on depressive symptoms. An effect of EMA on depressive symptoms would limit EMA as an instrument, because a change in EMA ratings might be attributed to the EMA itself instead of treatment or other factors. This is of immediate interest to research projects in which participants carry out EMA over longer periods, such as the E-COMPARED project [8]. However, a positive effect would indicate that EMA can be applied as an intervention, e.g. alongside psychotherapy or pharmacotherapy. A negative effect would indicate that it may not be ethical to use EMA as an instrument. If we find indications of response fatigue at T6 and/or T12, we can recommend the optimal period of conducting EMA.

### Limitations

First, a change in retrospectively measured depressive symptoms could be attributed not only to a change in depression severity, but also to a change in response behaviour. If we find an effect, we can explore changes in response behaviour by testing the CES-D outcomes for measurement invariance [28]. Second, one of our secondary aims is to investigate response rate, but the response rate might be artificially high, because participants are given a monetary reward when they complete 80 % or more of the EMA prompts. This reward is necessary to answer the primary research question, because any effect of EMA can only be shown when participants conduct EMA.

### Conclusion

To our knowledge, this is the first study to investigate whether self-rated EMA of depressive symptoms could induce assessment reactivity among mildly depressed individuals.

## Abbreviations

CES-D: Centre for epidemiologic studies depression scale
EMA: Ecological momentary assessment
PHQ-9: Patient health questionnaire
SUS: System usability scale
